# Large and transient positive temperature anomalies in Washington’s coastal nearshore waters during the 2013–2015 northeast Pacific marine heatwave

**DOI:** 10.1371/journal.pone.0280646

**Published:** 2023-02-01

**Authors:** Julie Ann Koehlinger, Jan Newton, John Mickett, LuAnne Thompson, Terrie Klinger

**Affiliations:** 1 School of Marine and Environmental Affairs, University of Washington, Seattle, Washington, United States of America; 2 Applied Physics Laboratory, University of Washington, Seattle, Washington, United States of America; 3 School of Oceanography, University of Washington, Seattle, Washington, United States of America; Universidade de Aveiro, PORTUGAL

## Abstract

The northern portion of Washington’s outer coast—known locally as the Olympic coast—is a dynamic region characterized by seasonal upwelling that predominates during summer interrupted by occasional periods of downwelling. We examined spring-to-fall water temperature records collected along this coast from 2001–2015 from April to October at four nearshore locations (Cape Elizabeth to Makah Bay) that span one degree of latitude and are located within 15 km of the shore. When compared against a long-term climatology created for 2001–2013, seven-day smoothed temperature anomalies of up to 4.5°C at 40 m depth during 2014 and 2015 show short-term warm events lasting 10–20 days. These periods of warming occurred within the well documented marine heatwave in the Northeast Pacific and were about twice the seasonal temperature range in the climatology at that depth. These warm events were strongly correlated with periods of northward long-shore winds and upper ocean currents, consistent with what is expected for the response to downwelling-favorable winds. While our focus *a priori* was on 2014 and 2015, we also found large positive temperature events in 2013, which were potentially related to the early stage of the marine heatwave, and in 2011, which did not have a documented marine heatwave. This indicates that near-shore short-term warm events occur during periods of large-scale offshore marine heatwave events, but also can occur in the absence of a large-scale marine heatwave event when downwelling-favorable winds occur during the summer/early fall.

## Introduction

Marine heatwaves (MHWs) are anomalous, large-scale warm water events characterized by their intensity, duration, and spatial extent [1, 2]. They have been reported from diverse locales, including the Mediterranean Sea [3], the north Atlantic [4, 5], the coast of western Australia [6], and the northeast Pacific [7, 8]. These events have been observed to cause geographic shifts in species distributions and declines in species abundance [9–12]. Notably, anthropogenic warming of the oceans is projected to increase the frequency, duration, intensity, and spatial extent of MHWs worldwide [13]. A MHW is generally defined as a local, sustained period of sea surface temperature (SST) that exceeds a threshold defined by a long-term climatology, typically at the 90% level [1]. Oliver et al. [14] show that from 1925–2016, MHW frequency, duration, and number have all increased owing to long-term warming and that we can expect further increases in the years ahead.

The impact of MHWs on local ecosystems depends in part on the ability of the ecosystem to adapt to long term warming [15]. Analysis of a coupled climate model with 10km ocean model resolution shows that within Large Marine Ecosystems, the threat to the organisms from MHWs will increase even if the ecosystems are able to adapt to long-term warming [16, 17]. This includes the California Current Large Marine Ecosystem that encompasses the Washington Shelf that we focus on here.

A major northeast Pacific MHW evident during 2013–2015 was associated with a pool of anomalously warm SST that spread over a large area including the California Current System (CCS) [7]. Di Lorenzo and Mantua [18] describe its development in the northeast Pacific during the autumn of 2013 and its persistence through 2015. Analysis of climate model simulations suggest that changes in the tropical Indo-Pacific Ocean likely drove atmospheric teleconnections that created this event [19]. In addition, coupled climate models also suggest a two-way coupling between tropical Pacific SSTs and the North Pacific [19].

More recent analysis of the same MHW offshore using Argo profiling floats [20] reveals a genesis in mid-2013 and persistence through 2016 as well as sustained subsurface warming down to 150 m that lags the warmest SSTs by about 60 months. Here, we focus on signatures of the northeast Pacific MHW of 2014–2015 (NEP 2014–2015 MHW) in the nearshore environment. During this event, offshore satellite sea surface temperature (SST) anomalies as large as +4°C were observed in the CCS [21], and continental shelf buoy data off La Push, WA, recorded positive temperature anomalies of more than 6°C mid-depth [22]. Coincident with this MHW, an El Niño developed in the equatorial Pacific in 2015 that may have added to its warming effect [19].

The impacts of this event on marine life were profound, including large die-offs of seabirds [23, 24], and a large persistent harmful algal bloom that extended from California to the Gulf of Alaska [25]. While the open-ocean characteristics of the MHW can be examined by satellites and profiling floats [20], the nearshore expression of the heatwave—that is, in waters of 60 meters depth or less—has remained relatively unexplored for locations in the northern reaches of the CCS, including those along Washington state’s Olympic coast.

We focus here on waters along the coast of Washington. This region provides socio-economic and cultural benefits to coastal communities, supporting valuable commercial, recreational, and subsistence fisheries, and is home to four federally recognized tribes—the Makah, Quileute, and Hoh Indian Tribes and the Quinault Indian Nation, all of which retain treaty-reserved rights to fish in the region. Moreover, the area is designated as a national marine sanctuary, with attendant protections for the marine resources within its boundaries. Understanding the nearshore expression of the NEP 2014–2015 MHW along the Olympic coast fills a conspicuous information gap, lending insight into the nearshore conditions that the local ecosystem experienced during this large event.

**Fig 1:**
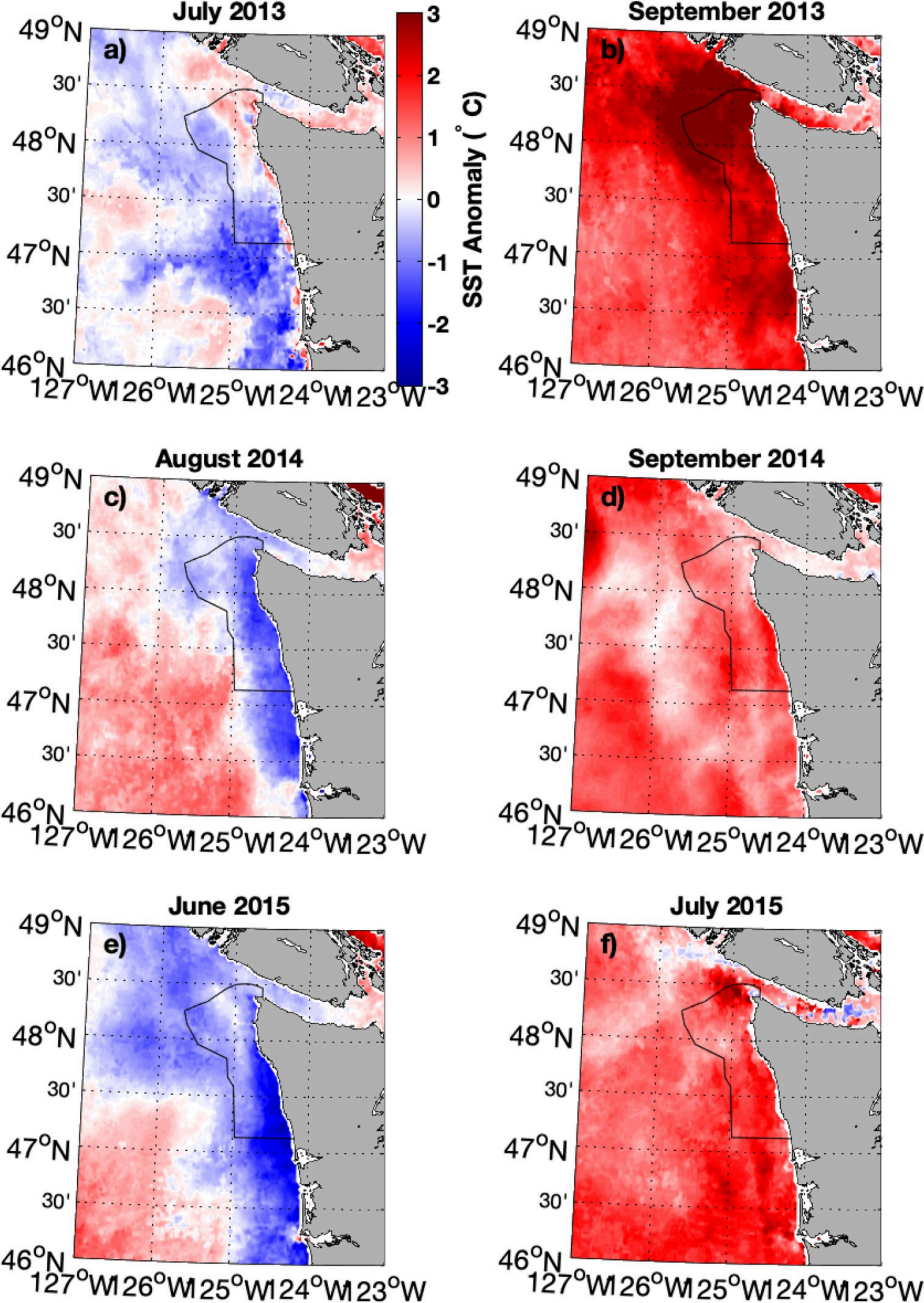
Monthly-averaged MODIS SST anomaly. Comparison of predominantly upwelling conditions (panels a, c, e) to downwelling conditions (b, d, f) for three different years during the 2013–2015 MHW. Upwelling more than offsets the MHW on the shelf, with anomalously warm water restricted to off-shelf waters during these periods. During downwelling conditions, anomalously warm SST extends to the coast.

The physical oceanographic characteristics of this and other eastern boundary current systems are modulated by the dynamics of seasonal upwelling and downwelling. Seasonal upwelling, which moves colder, saltier water upwards, occurs during the summer months on the Olympic coast, creating cold surface conditions relative to those offshore. Monthly-averaged satellite data showed that anomalously warm waters associated with the NEP 2014–2015 MHW were held offshore during periods of upwelling-favorable winds, and that positive temperature anomalies extended to the shore when the monthly averaged winds switched to downwelling-favorable [26]. Fig 1 showing MODIS Aqua SST anomaly (climatology July 2002—June 2013) demonstrates this evolution.

Here we examine the nearshore expression of the NEP 2014–2015 MHW in the broader context of seasonal upwelling in the very near shore environment, within approximately 15 km of the shore. To do this we constructed a spring-to-fall daily temperature climatology using data collected from 2001–2013 over the period from June 1- October 15, when upwelling is most likely to occur. We then examined 2014–2015 anomalies from this climatology to determine the impact of the NEP 2013–2015 MHW on the nearshore environment. Next, we examined the relationship of the nearshore wind-stress to the temperature anomalies to determine whether warming of the nearshore waters was associated with downwelling-favorable winds.

## Methods

**Fig 2:**
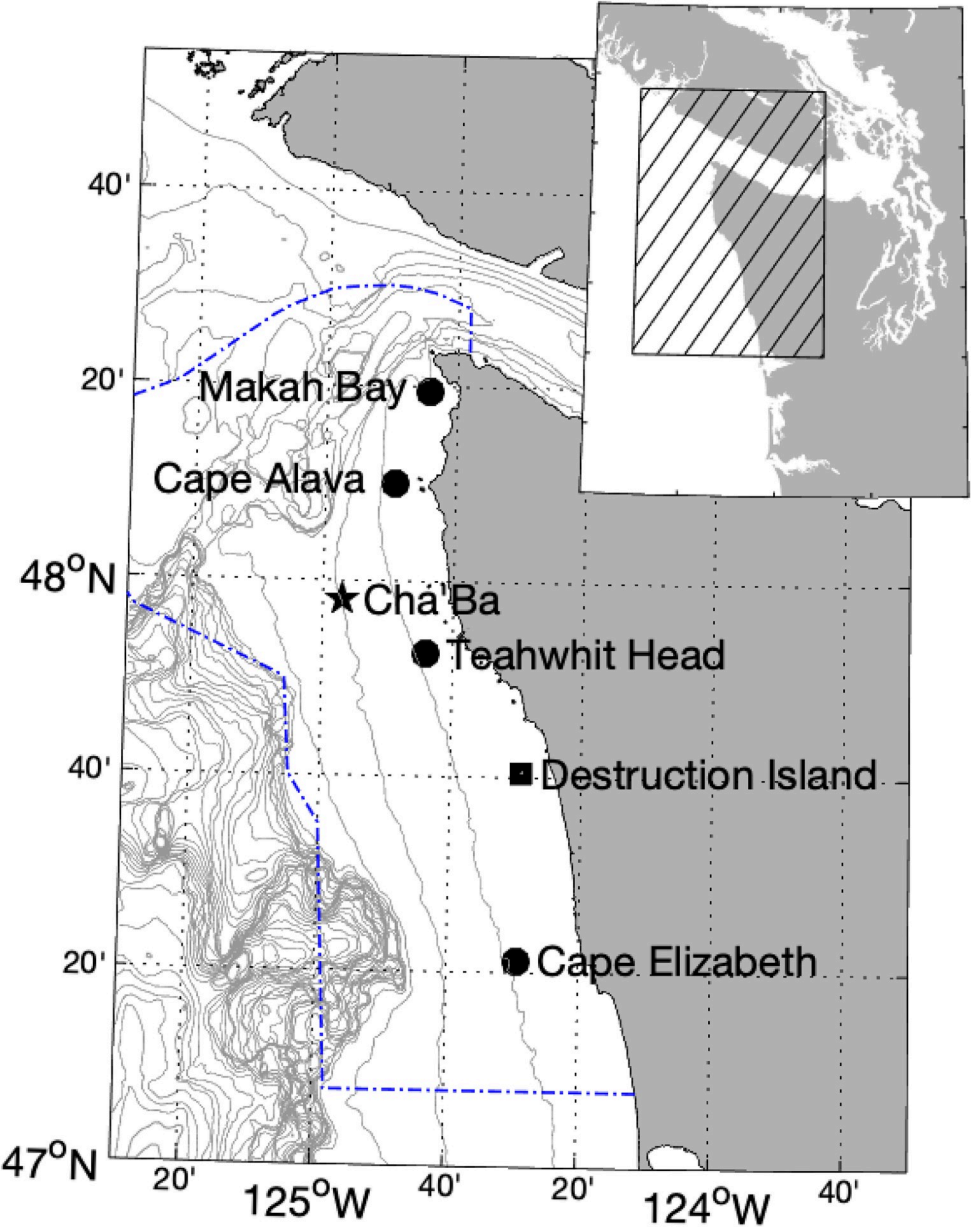
Mooring and meteorological station locations. OCNMS moorings include Makah Bay, Cape Alava, Teahwhit Head, and Cape Elizabeth. Meteorological data were obtained from the Destruction Island Station. Current data were obtained from the Cha’ba and subsurface buoys off of La Push. The OCNMS is outlined in blue.

We first constructed climatologies of subsurface (40 m) temperature at four moorings seasonally maintained by the Olympic Coast National Marine Sanctuary (OCNMS) along the Washington coast: Makah Bay (MB), Cape Alava (CA), Teahwhit Head (TH), and Cape Elizabeth (CE) (Table 1 and Fig 2). MB and CA moorings have been deployed since 2001. TH was added in 2002, and CE was added in 2004. All stations are within 15 km of the shore with bottom depths of approximately 42 m. The moorings were deployed from spring to fall each year.

**Table 1:** Locations and water depth of moorings and meteorological stations used in this study.

Station	Organization	Latitude	Longitude	Total Depth
Makah Bay (MB)	OCNMS	48.324 N	124.735 W	42 m
Cape Alava (CA)	OCNMS	48.166 N	124.823 W	42 m
Teahwhit Head (TH)	OCNMS	47.876 N	124.733 W	42 m
Cape Elizabeth (CE)	OCNMS	47.353 N	124.489 W	42 m
Destruction Is. (Station DESW1)	NOAA NBDC	47.675 N	124.485 W	n/a
La Push (Cha’ba & Subsurface)	NANOOS/UW-APL	47.97 N	124.95 W	100 m

The beginning and end dates for data collection varied by year and by station. OCNMS’s seasonal oceanographic moorings within the sanctuary are typically deployed near the end of May and recovered sometime in October, depending on local weather conditions. For consistency, we restricted our temperature analysis to data collected between June 1 and October 15. Equipment failure led to occasional gaps in the record and the absence of data from the Teahwhit Head mooring in 2011. Instrumentation varied by year and included the Onset StowAway TidbiT Temperature Data Logger TBI-32, the Onset TidbiT v2 Water Temperature Data Logger UTBI-001, and the Sea-Bird Electronics 37-SM MicroCAT and SeaCAT 16 plus. Sampling frequency was every 2, 8, or 10 minutes. Detailed temperature and acoustic doppler current profiler (ADCP, discussed below) instrumentation information is listed in Table 2.

**Table 2:** Instrumentation used by year, station, depth, and sensor accuracy.

Instrumentation	Years	Station	Depths	Accuracy
TidbiT TBI-32	2001–2005	MB	40 m	+/- 0.2°C
		CA		
	2002–2005	TH		
	2009 (May 1-July 8)			
	2010 (June 6-August 20)			
	2004–2006	CE		
TidbiT	2009 (July 9-October 7)	TH	40 m	+/- 0.02°C
UTBI-001	2010 (August 21-October 12)			
	2014–2015	MB	near-surface, 5, 10, 20, 30, 40 m	
		CA		
		TH		
		CE		
SBE 37-SM MicroCAT	2006–2011	MB	40 m	+/- 0.002°C
SBE SeaCAT	2012–2015	TH	40 m	+/- 0.002°C
16-plus		MB		
	2006–2015	CA		
	2007–2008	TH		
	2012–2015			
	2007–2015	CE		
Teledyne RDI ADCP: 300 kHz, upward-facing	2014 (August 2 –October 7)	NANOOS-Subsurface	7 to 85 m in 2-m bins	< |0.02| m s^-1^
Teledyne RDI ADCP: 600 kHz, upward-facing	2014 (June 25 –September 21)	NANOOS-Subsurface	3 to 15 m in 0.5-m bins	< |0.02| m s^-1^
	2015 (May 23 –October 22)			

We examined temperature anomalies at 40 m nominal depth by first constructing daily climatologies for the 10- to 13-year period up to and including 2013. The analysis focused on data from 40 m depth to minimize influence from local transient atmospheric forcing that may affect surface values. The data within each 24-hour period were averaged to create daily means. Climatology was determined by first averaging daily values over the baseline period and then smoothing this composite year using a moving seven-day window. In order to specifically investigate the anomalies through the water column during 2014 and 2015, data from fixed depth sensors on each mooring at nominal depths ranging from 1–40 m were also smoothed using a moving seven-day window. We derived temperature anomalies at 40 m for 2014–2015 by subtracting the climatology from these smoothed absolute temperatures.

To investigate the potential influence of local winds on the timing, evolution and magnitude of temperature anomalies, we used wind data from NOAA’s NBDC Destruction Island Station (Table 1 and Fig 2) for the period June 1-October 15 in 2014 and 2015. NOAA’s wind data was a 60-minute average of 2-minute sample periods. We rotated wind direction 15 degrees counterclockwise to align with the coast and the along-shore northward component of wind stress was calculated following Large and Pond [27]. Wind stress was smoothed with a boxcar filter over 24 hours, linearly interpolated onto a daily grid, and then smoothed with a moving seven-day average.

We used current data from the Northwest Association of Networked Ocean Observing Systems (NANOOS) / University of Washington Applied Physics Lab’s (UW-APL) subsurface mooring off of La Push, Washington (Table 1 and Fig 2) to further investigate mechanisms driving temperature variations. Current data from August 2 –October 7, 2014 were obtained from a 300 kHz upward-facing ADCP deployed at a nominal depth of 90 m. Water profiling pings collected at 25-second intervals were averaged into 10-minute ensembles at 2 m depth intervals. Current data from June 25 –September 21, 2014 and May 23 –October 22, 2015 were obtained from a 600 kHz upward-facing ADCP at a nominal depth of 15 m. Ten 1-second water profiling pings were averaged into 2-minute ensembles at 0.5 m depth intervals, and subsampled every 10 minutes. We used average current data from 8–10 m depth, which was approximately the center of the measurement depth range (~3-15m) and well within the wind-driven surface layer for most time periods. Data from both years were smoothed over 24 hours with a boxcar filter, and linearly interpolated onto a daily grid. Data were then smoothed with a moving 7-day average for comparison with wind data.

## Results

### 2001–2013 climatology

**Fig 3:**
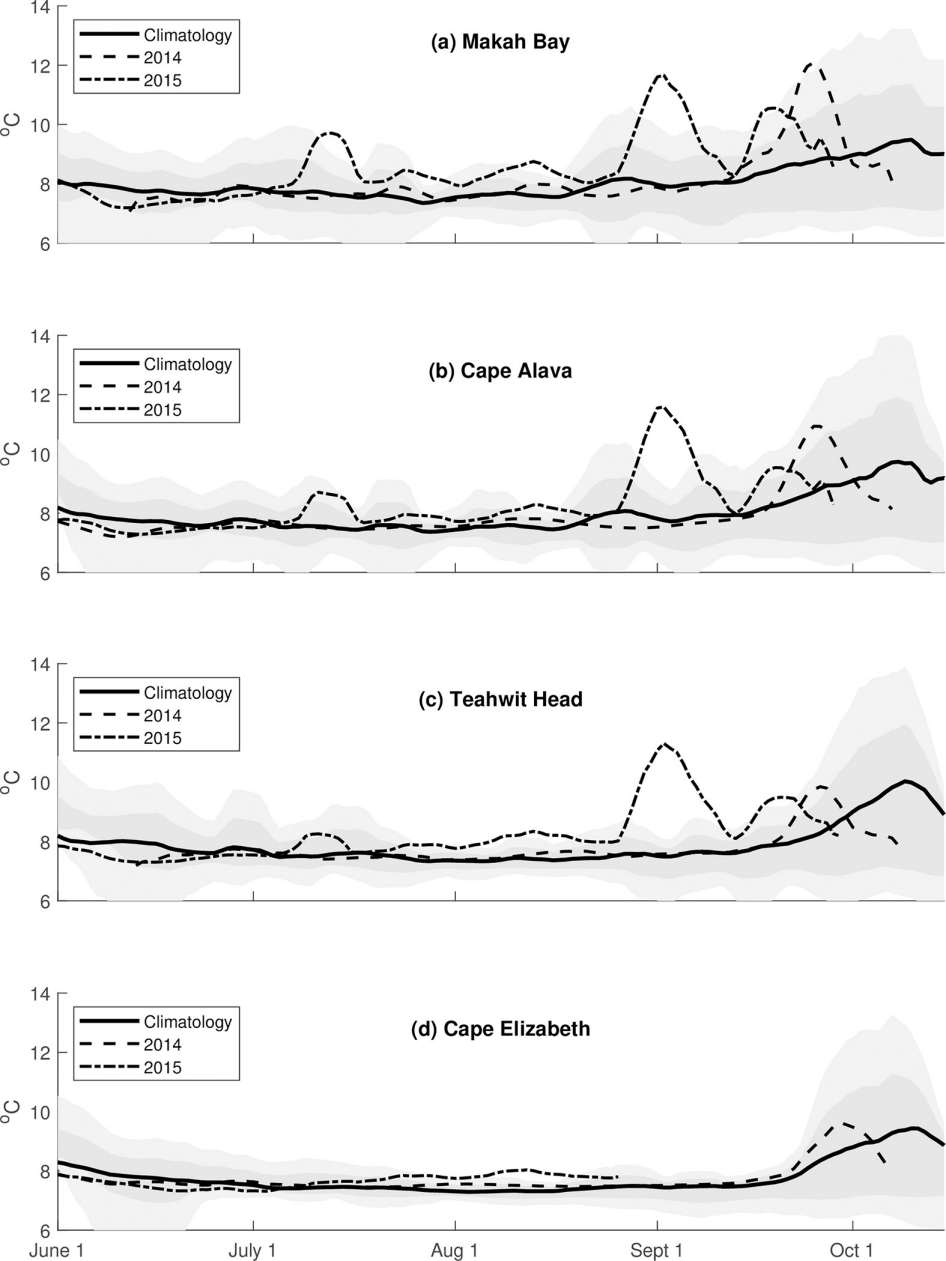
Climatology and observed 40 m temperatures in 2014 and 2015. Climatology (solid line) and observed 40 m temperatures in 2014 (dashed line) and 2015 (dash-dot line) at (a) Makah Bay, (b) Cape Alava, (c) Teahwhit Head, and (d) Cape Elizabeth with +/- one standard deviation (darker gray shading) and +/- two standard deviations (lighter gray shading).

At all locations, cooling occurred in late spring (June), with colder waters throughout summer that became warmer during fall (late Sept; Fig 3). The maximum temperature over the June 1 to October 15 period occurred after October 1, and late summer/early fall temperatures showed greater variability in their range compared to those in spring and summer (Fig 3 and Table 3). Minimum temperatures were similar across the four stations (7.3–7.4°C), while the range in the maximum temperatures at the four locations was larger (9.4–10.1°C) (Table 3). Even so, maximum temperatures at all stations remained within one standard deviation of each other. The maximum temperature increased from north (MB) to south (TH), with the exception of CE, the southernmost station, which was colder than any of the other stations.

**Table 3:** Minimum and maximum 40 m temperatures (1 June– 15 October) at each station with +/- 1 standard deviation.

Station (years of climatology)	Minimum (°C)	Maximum (°C)
Makah Bay (2001–13)	7.3 +/- 0.3	9.5 +/- 1.9
Cape Alava (2001–13)	7.4 +/- 0.3	9.7 +/- 2.2
Teahwhit Head (2002–13)	7.4 +/- 0.2	10.0 +/- 2.1
Cape Elizabeth (2004–13)	7.3 +/- 0.1	9.4 +/- 1.6

Stations are listed in order from north to south. Initial year of data collection varies as noted.

### 2014–2015 temperature anomalies

The 40-m records from 2014–2015 were characterized by temperatures significantly warmer than climatology, showing warm water events at MB, CA, and TH that persisted for 10–20 days. Moreover, the maximum temperature during this period was *highest* at MB and *decreased* from north to south, opposite to what was found in the climatology. In most cases there was a north-south gradient in the magnitude of the positive temperature anomalies, with the largest positive temperature anomalies in the north at MB and the smallest in the south at CE (Table 4).

**Table 4:** Maximum temperature at 40-m, maximum temperature anomaly at 40-m, and number of standard deviations above climatology at each station during peak temperature anomaly periods.

Station	Sept 2014	Early Sept 2015
	Temperature (°C)	Temperature (°C)
**Makah Bay**		
Maximum	12.1	11.7
Max. Anomaly	3.4	3.7
Std. dev. (σ) above climatology	2.2	4
**Cape Alava**		
Maximum	10.9	11.6
Max. Anomaly	1.5	3.8
Std. dev. (σ) above climatology	1.5	6.5
**Teahwhit Head**		
Maximum	9.9	11.3
Max. Anomaly	1.6	3.8
Std. dev. (σ) above climatology	1.4	13.2
**Cape Elizabeth**		
Maximum	9.6	no data
Max. Anomaly	1.0	n/a
Std. dev. (σ) above climatology	<1	n/a

Values were calculated using the 7-day smoothed and daily interpolated observations for the two peak anomaly periods observed in each year’s record (1 June-15 October).

Positive temperature anomaly pulses were seen throughout 2014–2015 but were largest during the late summer/early fall (September) and were close to 4°C during both years (Fig 3). Plots of the unsmoothed daily anomalies for these years (not shown) indicates that these events last for 5–10 days.

**Fig 4:**
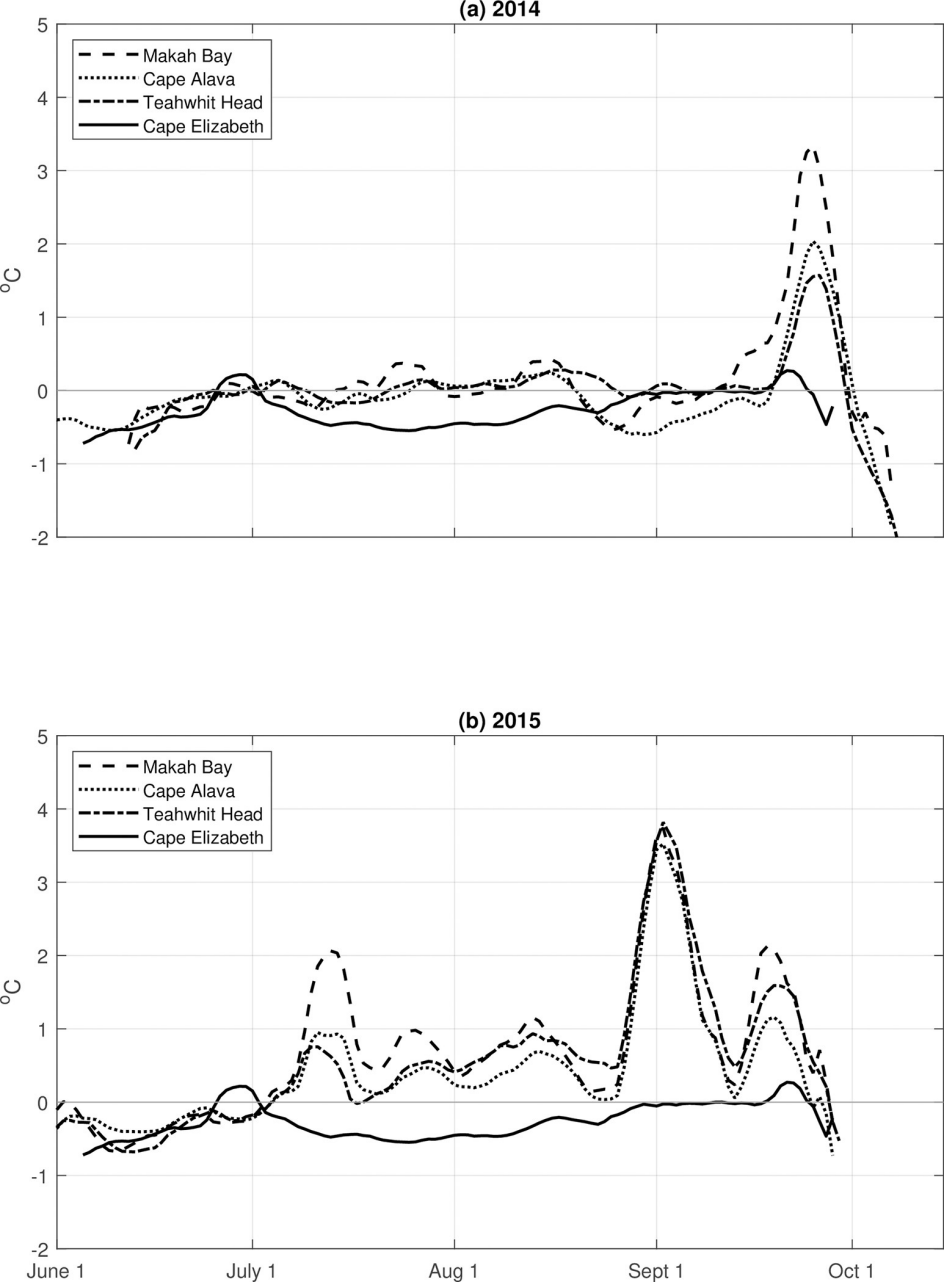
Temperature anomalies in 2014 and 2015. Temperature (40 m) anomalies in (a) 2014 and (b) 2015 at Makah Bay (dashed line), Cape Alava (dotted line), Teahwhit Head (dash-dot line), and Cape Elizabeth (solid line).

In 2014, 40-m temperatures remained within +/- 1 standard deviation of the climatology at all stations until late September. The largest anomaly (3.5°C) was observed at MB in the north and there was a north-south gradient with an anomaly of only +1°C (<1 standard deviation) at CE (Table 4 and Fig 4A).

Three positive temperature anomalies were observed in 2015, with one occurring in July and two in September. Two smaller anomalies (~1°C) are also observed at Makah Bay in late July and mid-August. The July and second September peaks followed the same spatial pattern as in 2014 were of similar magnitude (3.8–4.0°C). There were no data available for CE after mid-August (Table 4 and Fig 4B). The early September temperature anomalies at MB, CA, and TH are substantially above 2 standard deviations, showing 4, 6.5, and 13.2 standard deviations above climatology, respectively (Table 4). While we recognize that the fourteen-year record may not have a Gaussian distribution, we report the standard deviation to indicate the relative ranking of the excursions during the MHW.

### Year to year variation

Large positive temperature anomalies were also present outside of the NEP 2013–2015 MHW years, notably 2011. There was no offshore NEP MHW signature in 2011, only a small band of nearshore warmer-than-average waters seen in MODIS satellite data (not shown). This implies that there are other mechanisms capable of generating large positive temperature anomalies, such as strengthening of downwelling-favorable winds.

**Fig 5:**
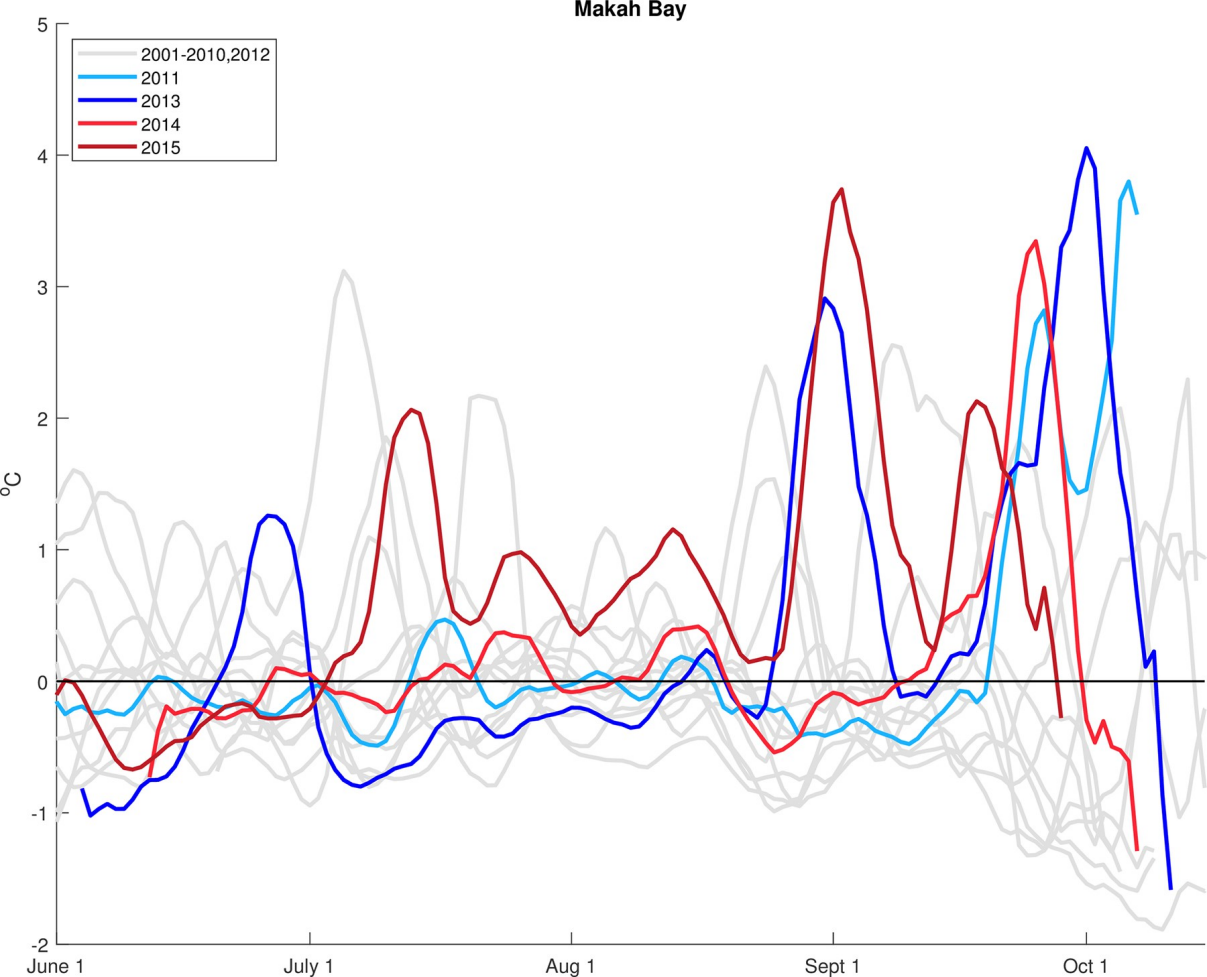
Temperature (40 m) anomalies at Makah Bay for each year from 2001 to 2015. Years with large warm events are plotted in colors.

The appearance of nearshore positive temperature anomalies is most apparent at Makah Bay (Fig 5). In each year examined, all but one of the largest anomalies occurred during the fall (after mid-August) and were consistent with the much larger standard deviation from the climatology during this time period.

#### Vertical extent

**Fig 6:**
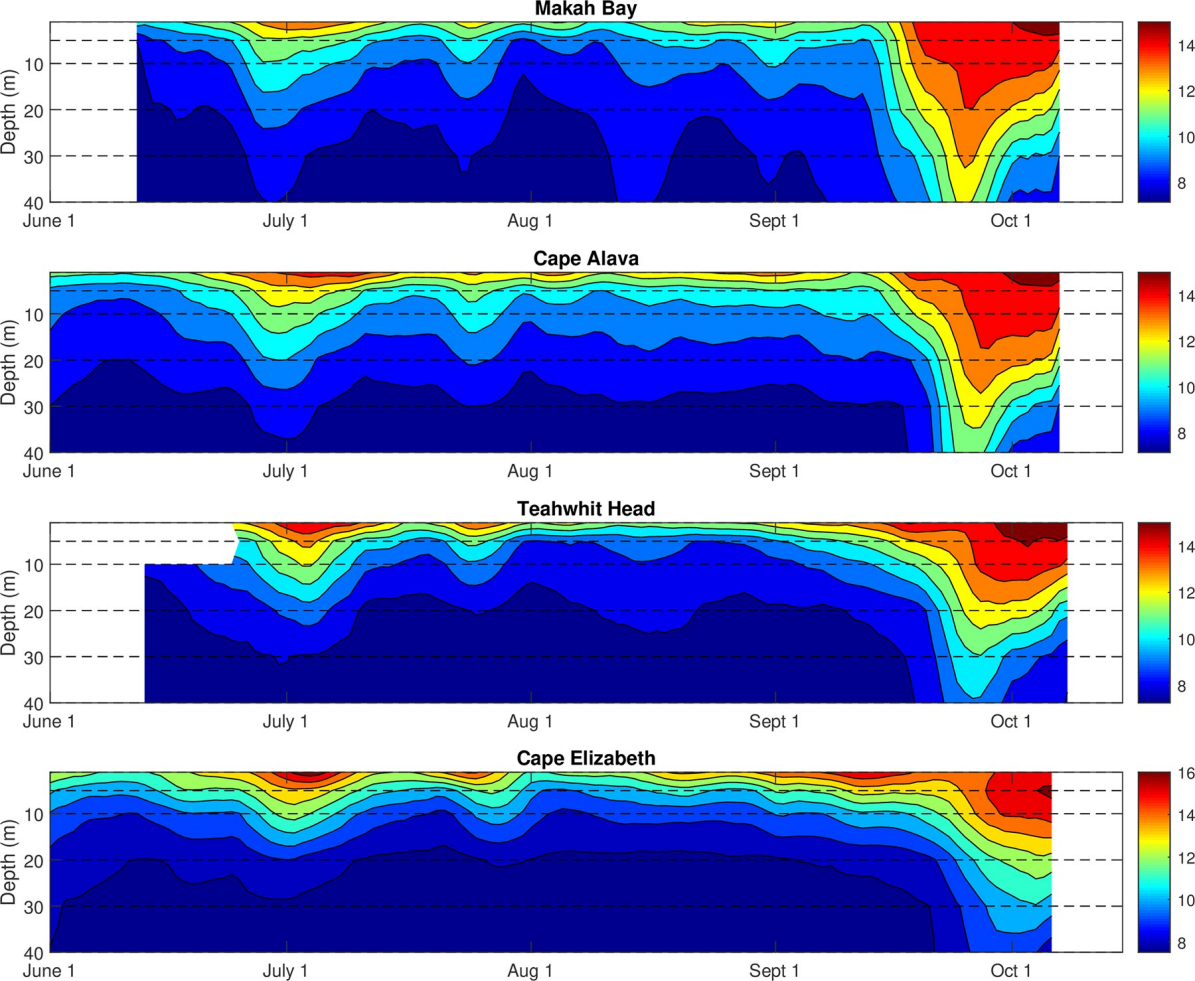
Depth-time contours of temperature in 2014 at (a) Makah Bay, (b) Cape Alava, (c) Teahwhit Head, and (d) Cape Elizabeth. Dashed lines indicate approximate depths of instruments.

**Fig 7:**
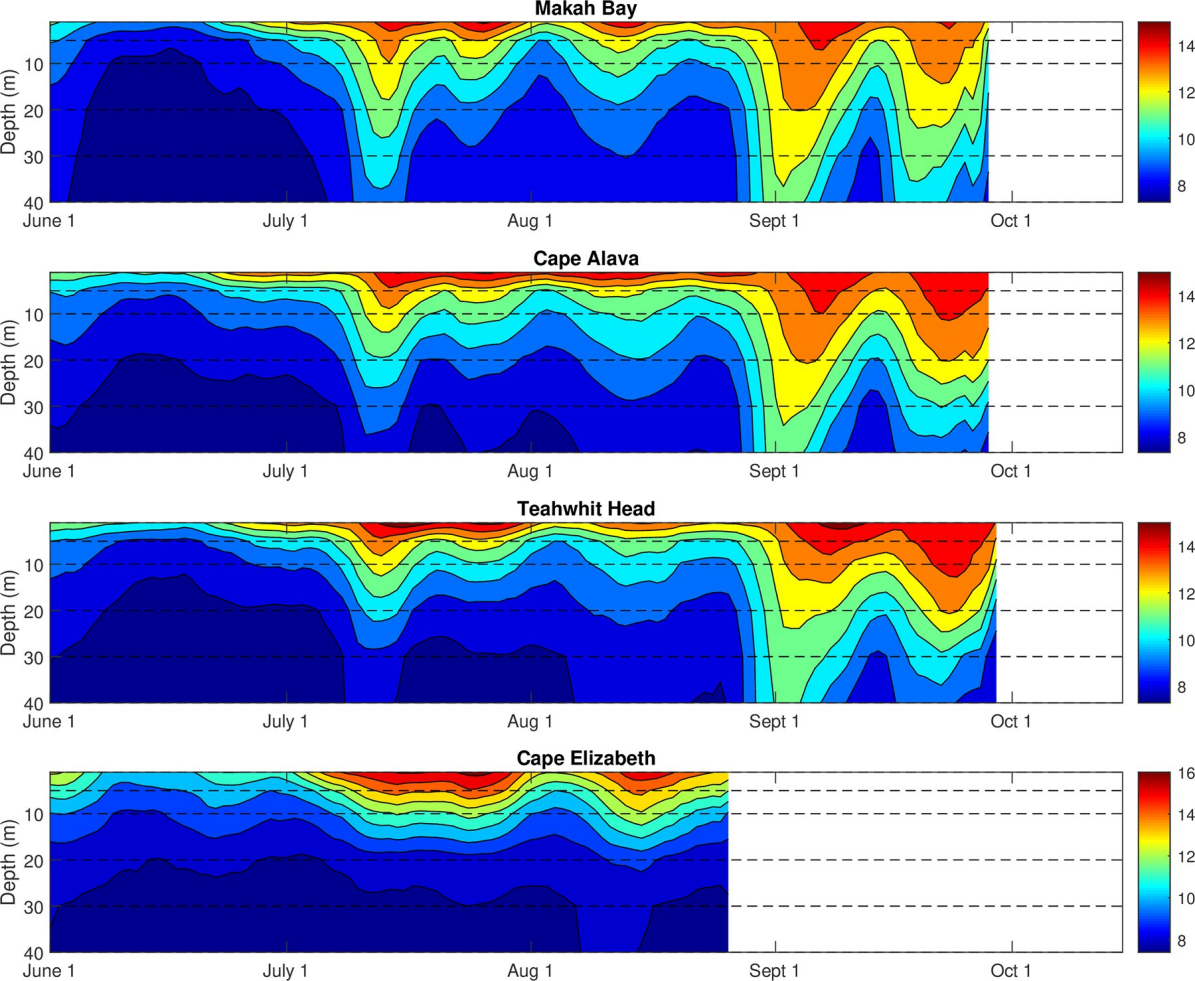
Depth-time contours of temperature in 2015 at (a) Makah Bay, (b) Cape Alava, (c) Teahwhit Head, and (d) Cape Elizabeth. Dashed lines indicate approximate depths of instruments.

Positive temperature anomalies were observed throughout the water column, as shown by data taken from near surface to 40 m depth (Figs 6 and 7). The dynamics and timing of the positive temperature anomalies varied with depth. Generally, changes in temperature nearer to the surface lagged changes in temperature at depth, although this was not always the case.

In July 2014, warming occurred at all depths at approximately the same time. However, in September 2014, temperatures at 20, 30, and 40 m peaked at about the same time and subsequently declined while the near-surface, 5 m, and 10 m temperatures continued to increase and then stabilize for another week before beginning to decline. This pattern was observed at all stations except Cape Elizabeth, where only the 40 m temperature record shows an earlier peak and decline (Fig 6). The warming reaches a maximum at the surface at all stations except Cape Elizabeth in October.

In 2015, warming in July was followed by two distinct warming events in September (Fig 7). During the early September event, the temperature at Makah Bay and Cape Alava at 30 m and 40 m increased and then cooled before that of the near-surface temperature. Cooling moved upward in the water column with about a 2-day lag between each 10 m change in depth giving an implied upward velocity of 5 m/day. Teahwhit Head showed a similar 2-day lag from 30 m and 40 m to 20 m; however, the 5 m and near-surface depths lagged the 20-m depth by about 5 days. No data were available for Cape Elizabeth beyond August 25 (Fig 7).

In contrast, in late September, the onset of warming began at 40 m across all stations and spread throughout the water column over 1–2 days, except for the near-surface temperatures at Makah Bay and Teahwhit Head, which lagged the other depths by an additional 2 days. Cooling occurred much more rapidly through the water column, beginning at 30–40 m and reaching the near-surface within 2–3 days (Fig 7).

### Correlation of positive temperature anomalies with winds and currents

**Fig 8:**
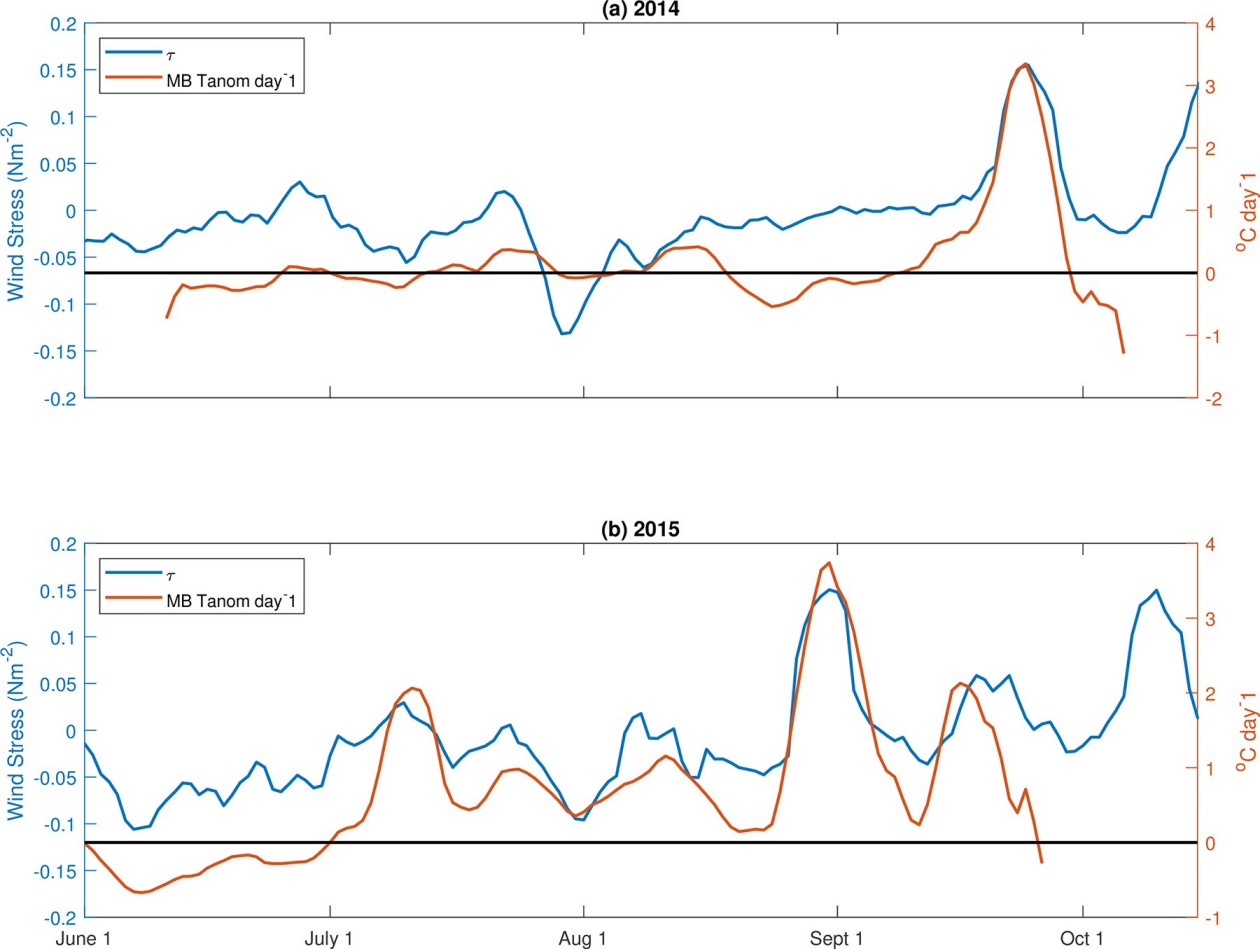
Alongshore wind stress at Destruction Island (blue line) and daily 40 m temperature anomaly at Makah Bay (orange line) in (a) 2014 and (b) 2015. Temperature anomaly lags wind stress by 1 day in 2014 and 2 days in 2015. Positive (negative) values indicate northward (southward) wind stress.

**Fig 9:**
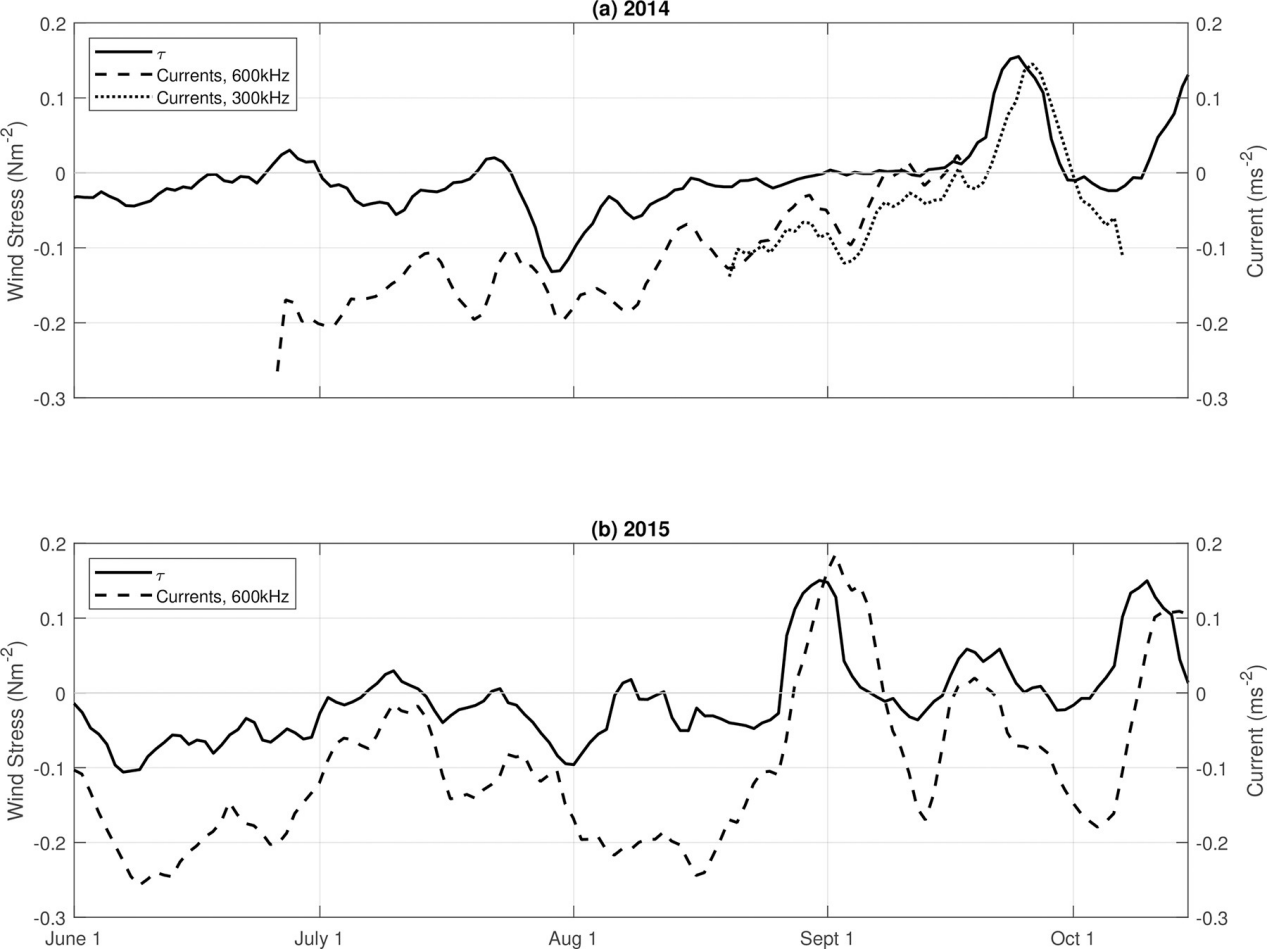
Wind stress at Destruction Island (solid line) and along-shore currents averaged over 8–10 m (dashed and dotted lines) in (a) 2014 and (b) 2015. Positive (negative) values indicate northward (southward) wind stress and along-shore currents Current data from 2014 taken from 600 kHz (dashed line) ADCP and 300 kHz (dotted line) ADCP.

Because alongshore wind stress is an important driving force of coastal dynamics in this region, we investigated the relationship between the positive temperature anomalies and along-shore wind stress using wind data from Destruction Island (located approximately 70 km south of Makah Bay) in 2014 and 2015. We found that the near-bottom temperature anomaly was significantly correlated with the along-shore wind stress (Fig 8) during both 2014 and 2015. In 2014, the correlation was strongest when temperature anomalies lagged wind stress by 1 day (r = 0.76). In 2015, this strong correlation was seen with an increased lag period of 2 days (r = 0.77). When along-shore winds were downwelling-favorable (winds from the south), temperature throughout the water column was typically elevated, particularly in the fall. Temperature was lower with upwelling-favorable winds (from the north). Along-shore currents at Cha’Ba follow the along-shore wind-stress, with a lag of several days (Fig 9), which may account for the response time for geostrophic adjustment as further discussed below.

## Discussion

**Fig 10:**
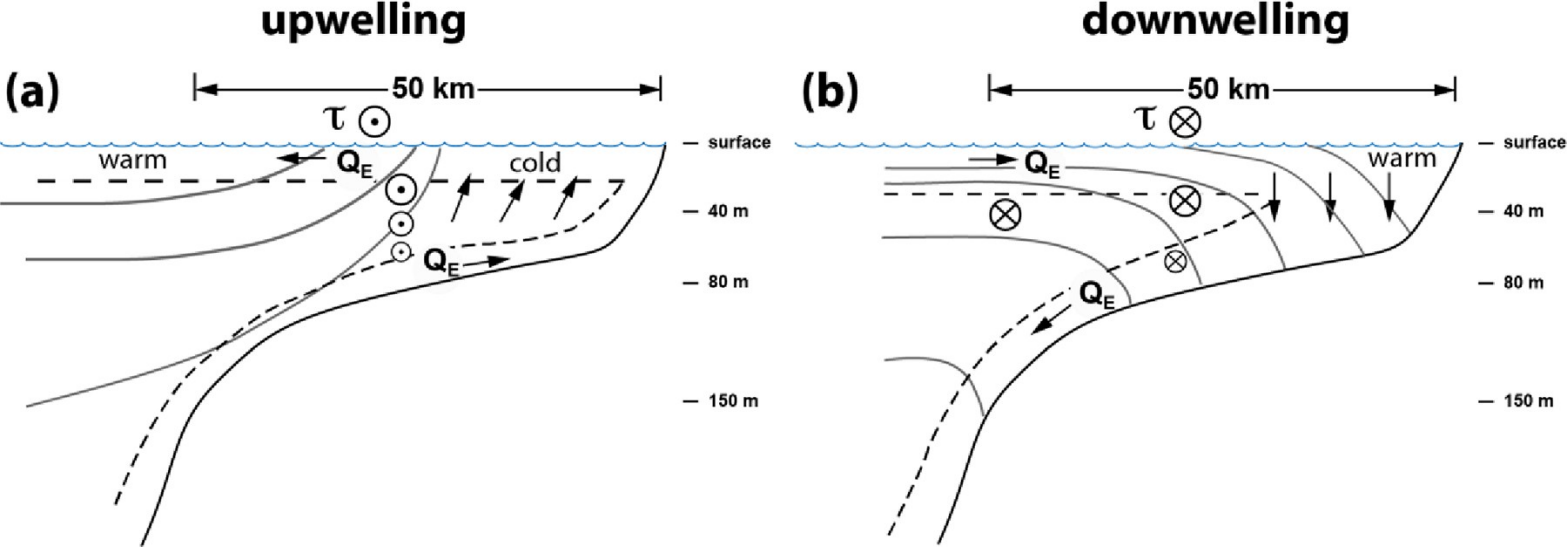
Schematic cross section of the Pacific Northwest shelf for (a) upwelling and (b) downwelling favorable along-shore wind stress, τ. Arrow heads (tails) show winds and currents from the north (south). Downwelling winds create shoreward Ekman transport (Q_E_), pushing warmer near-surface water inshore and downward, creating the positive temperature anomalies. Bottom Ekman transport can enhance upwelling and downwelling circulation.

It has been well-established that the dominant cross-shelf momentum balance of most inner-shelf regions, including the PNW, is geostrophic. As such, cross-shelf pressure gradients, largely driven by sea-surface slope and cross-shelf density gradients, balance the Coriolis acceleration (Coriolis parameter multiplied by along-shelf velocity) [e.g., 28]. For the *along-shelf* balance in the PNW, changes in depth-averaged along-shelf velocity are driven by both along-shelf wind stress and the along-shore pressure gradient [29], with contributions from Ekman flow when considering depth-dependent (baroclinic) flow [30]. With observed positive temperature anomalies associated with deeper isotherms (and isopycnals), we draw on this dynamical framework to hypothesize that the positive temperature anomalies result from poleward (northward) wind stress, poleward shelf flow, and isotherms (isopycnals) sloping downward toward the coast, or geostrophic adjustment to the poleward flow (Fig 10).

During upwelling conditions offshore, near-surface transport, onshore bottom Ekman transport, and resultant upwelling result in cooler water inshore. Flow is to the north and isotherms slope upward from offshore to inshore, satisfying the dominantly geostrophic cross-shelf momentum balance. Warmer near-surface water, which is influenced by local surface heating and pre-existing offshore conditions (such as heat waves), is found just off the shelf (Figs 1 and 10A). When upwelling winds relax or there is a switch to downwelling conditions, Ekman transport then moves this warmer, offshore, near-surface water inshore, pushing isotherms downward inshore due to negative wind stress curl (stronger northerly wind stress offshore) and the land boundary (Fig 10B). Figs 8 and 9, which show significant correlation between the warm anomalies and poleward along-shelf winds and along-shelf velocities, are consistent with this interpretation.

Guided by this hypothesized dynamical framework, to better understand the connection of the warm anomalies to Ekman dynamics we calculated the wind-driven Ekman transport (Q_E_, in m^2^s^-1^):

$$Q_{E}=\tau_{y}/f\rho$$

(where τ_y_ is the along-shore component of wind stress, *f* is the Coriolis frequency and *ρ* is the average density at CE). The transport, Q_E_, is 90 degrees to the right of the wind stress direction.

**Fig 11:**
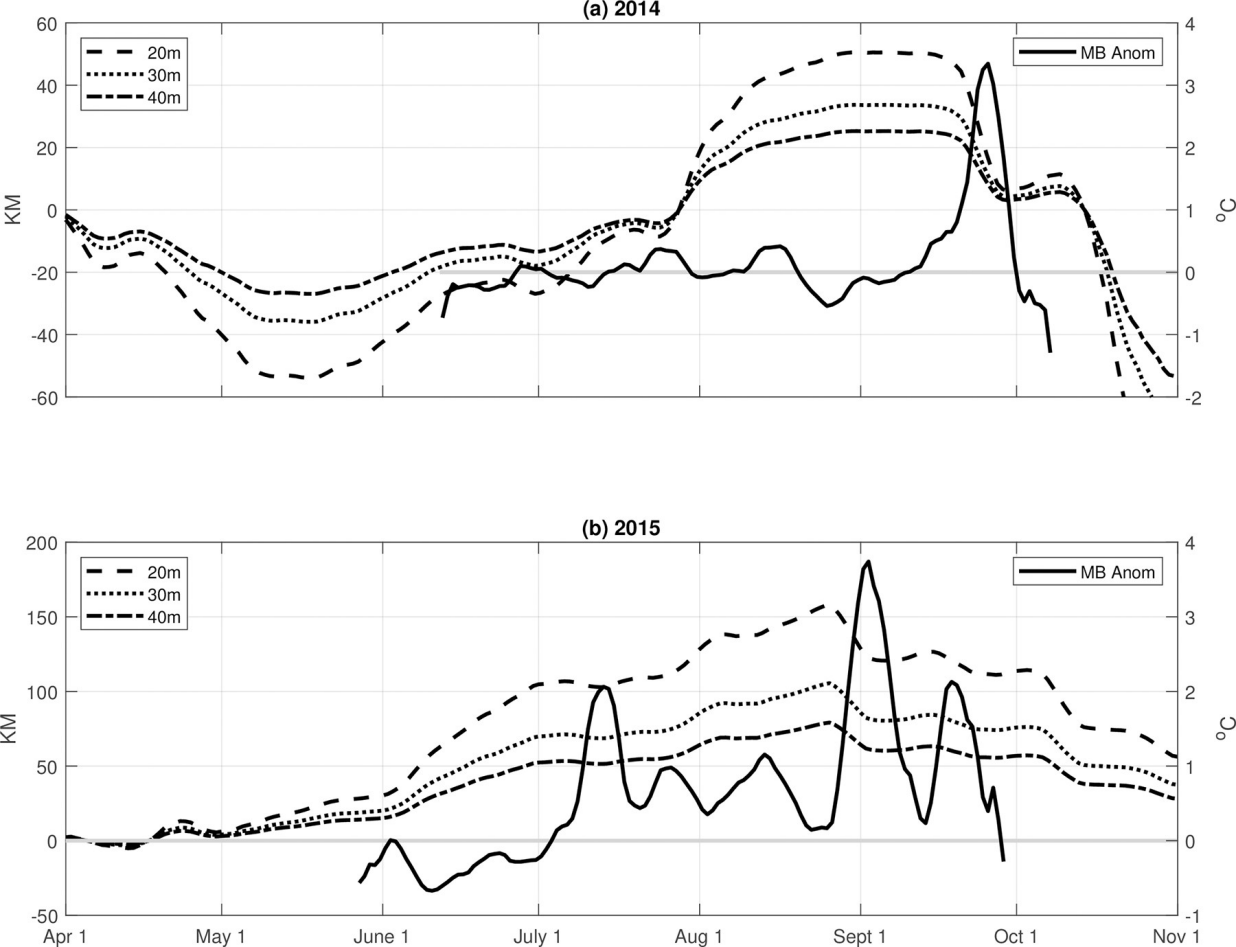
Cumulative travel from time-integrated Ekman transport in km, assuming Ekman layer depth of 20 m (dashed line), 30 m (dotted line), and 40 m (dash-dot line) in (a) 2014 and (b) 2015. Ekman transport in a positive (negative) direction indicates offshore (onshore) transport. Makah Bay temperature anomalies (solid line) are plotted with the right sided y-axis.

This analysis was motivated by our conceptual framework for the mechanism driving the positive temperature anomalies, which is a straightforward consequence of first-order coastal Ekman dynamics (Fig 11). Specifically, during upwelling conditions, offshore near-surface transport and upwelling result in cooler water inshore and isotherms sloping upward from offshore to inshore. Warmer near-surface water, which is influenced by local surface heating and pre-existing offshore conditions (such as heat waves), is found just off the shelf (Fig 11A). When upwelling winds relax or there is a switch to downwelling conditions, Ekman transport then moves this warmer offshore near-surface water inshore, pushing isotherms downward inshore due to negative wind stress curl (stronger northerly wind stress offshore) and the land boundary (Fig 11B).

Assuming a fixed Ekman layer depth, integrating this Ekman volume transport over time allows estimates of wind-forced transport cumulative *distance* in the offshore (onshore) direction due to upwelling (downwelling) winds. Using estimated Ekman depths of 20, 30, and 40 m to convert the Ekman transport to a velocity, in 2014 this transport could have moved fluid parcels a cumulative distance of 50–100 km offshore between mid-May and mid-September, at which point the Ekman transport changes sign, reversing transport to onshore (Fig 11A). In 2015, water could have moved a distance of 75–150 km offshore between May and late August (Fig 11B). Cumulative offshore transport peaked in late August 2015, just before the first large positive temperature anomaly in 2015, when there was again a change to downwelling-favorable winds and the direction of Ekman transport changed from offshore to onshore (Fig 11B). For both 2014 and 2015, positive temperature anomalies occurred when winds became downwelling-favorable and the direction of Ekman transport changed from offshore to onshore (Fig 11A and 11B). We infer that downwelling-favorable conditions advected warmer water shoreward, thus pushing the isotherms downward (Figs 6 and 7). The subsequent relaxation of downwelling-favorable winds allowed the isotherms to rebound upward. Estimated shoreward transport distances, however, were 40 km or less for each of the warm anomaly events, suggesting that much of the warm water was local shallow shelf water, and not water from off the shelf. Additionally, the temperature response to shifts in transport was fairly rapid (e.g., late August 2015, Figs 8 and 11), also indicating that the source water for the anomalies was local shelf water. Chan et al. [31] reported similar results for pH variability in Oregon and California. Again, these findings are largely consistent with modeled upwelling and downwelling on the inner continental shelf (e.g., [32, 33]).

**Fig 12:**
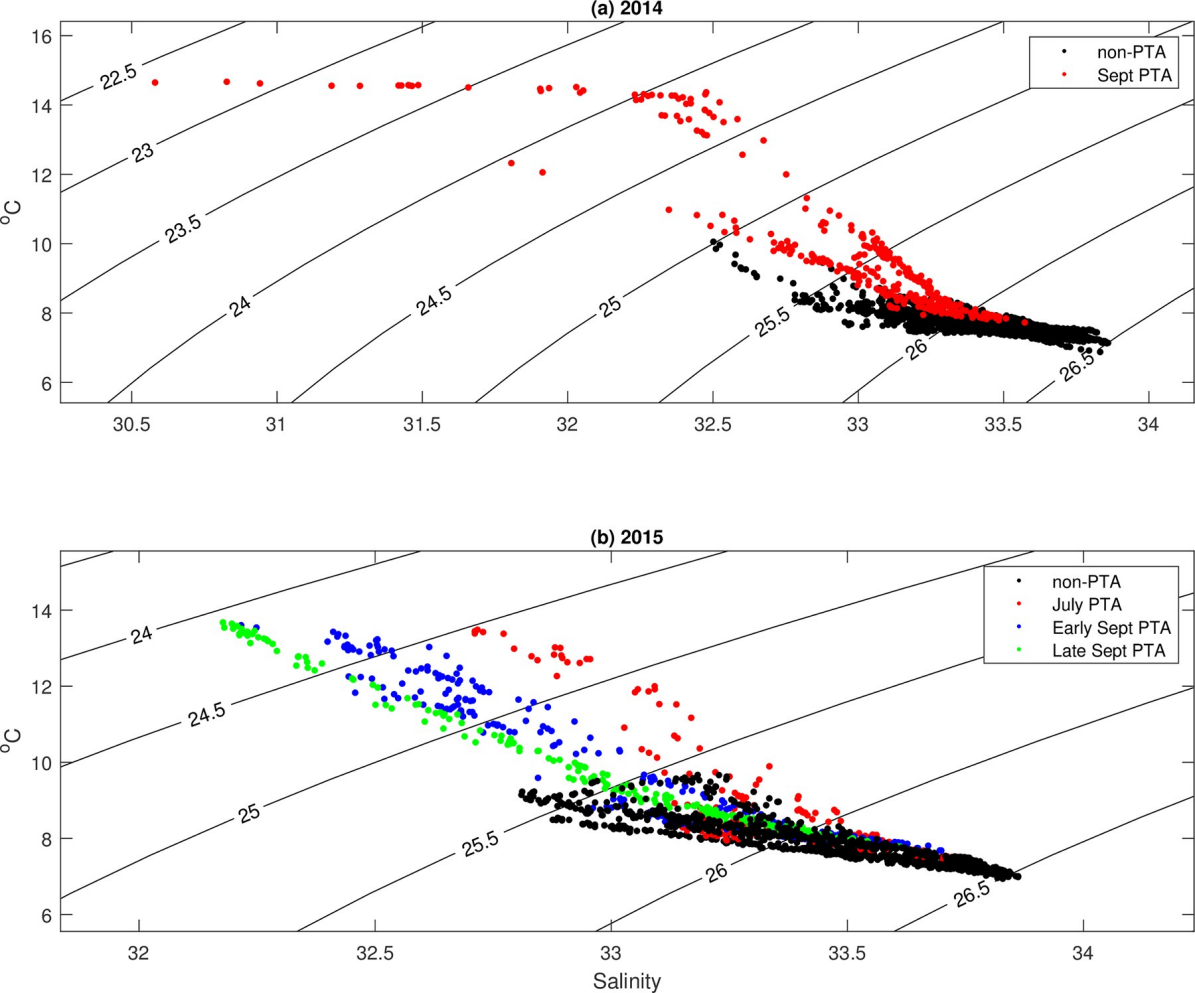
2014 (a) and 2015 (b) temperature versus salinity plots at 40 m nominal depth with lines of constant density. Warmer and fresher water is present during positive temperature anomaly events (colored dots) versus the non-event times (black dots).

Salinity variations are consistent with our interpretation of the causes of variations in temperature. At Makah Bay (Fig 12), when the temperature at 40m began warming, it also became fresher for the duration of the positive temperature anomaly, likely owing to both the cessation of upwelling that transports saltier water to the inner shelf and increased freshwater input via rain and rivers that is associated with strong southerly wind (storm) events.

The advection of an offshore water mass of uniform alongshore temperature into the nearshore would result in larger *anomalies* in the north, given that our reference climatology shows decreasing temperatures to the north (Table 3 and Fig 3) except for Cape Elizabeth. However, in 2014–2015 we detected not only a positive north-south anomaly gradient (Fig 4), but also a positive north-south absolute temperature gradient (Fig 3). That is, temperatures (at 40 m depth) in the north were warmer than temperatures in the south and consistently decreased from MB to CE during the summers of 2014 and 2015.

With the relaxation of downwelling-favorable winds and decrease in the northward along-shore velocity, cooling occurred throughout the water column and temperature anomalies at all stations returned to within +/- 1°C of the climatology. As noted earlier, temperature changes nearer to the surface generally lagged temperature changes at depth (Figs 6 and 7). There are several possible reasons for this. First, consistent with first-order Ekman processes, relatively well-mixed, near-surface warm water pools and deepens inshore during downwelling due to shoreward transport of warmer, near-surface water and convergence (Fig 10B). As downwelling winds relax, the initial offshore advection of this warm water could delay or inhibit near-surface cooling due to upwelling. Additionally, cooling of near-surface waters via upwelling could be buffered by strong summer shortwave (solar) surface heat fluxes and mixing of this heat downward. If this is the case, upon the switch from upwelling to downwelling conditions surface waters would likely not experience as much warming as deep waters, particularly because during downwelling events there may be some surface *cooling* due to lower air temperatures and windy and overcast conditions. The lag in the timing of the peak temperature anomaly with decreasing depth may be related to depth-dependent response times to changes in the along-shore and/or cross-shelf density gradients when wind forcing reverses.

Cross-shelf comparisons can be made between the nearshore OCNMS data and temperature data from NANOOS/UW-APL’s Cha’Ba mooring, which is positioned farther (roughly 25 km) offshore in 100 m water depth. During 2013, Cha’Ba recorded near-surface (3 m) temperature warming events in early July and early September [34] that were similar in timing to the observations from the OCNMS stations. The record, however, does not extend long enough to compare with the late September positive temperature anomalies recorded by the OCNMS moorings. During 2014, Cha’Ba data show a fall warming event, but unlike the OCNMS moorings that show significant positive temperature anomalies in late September, Cha’Ba temperature data did not show warming below 40 m until well into October [22]. This may indicate a cross-shelf dependence on the magnitude of the anomaly, with larger anomalies inshore, potentially due to greater convergence inshore. During 2015, Cha’Ba recorded higher deep temperatures (at 85 m) than in previous years, but these near-bottom temperatures remained below 10 ^**O**^C until November [35].

As expected, the climatology for temperature at 40m along the Olympic Coast is dominated by the seasonal patterns of wind-driven upwelling and downwelling. The spring transition along the Washington coast occurs, on average, on April 29 (+/- 29 days) [36] and is thus not captured by the data used in our analysis. However, the late-season climatologic pattern of increased variance is consistent with yearly observations of the fall transition and an average fall transition date of September 26 (+/- 20 days) [36].

We found that the magnitude of the spring-to-fall seasonal temperature range of the 40-m climatology was 2–3°C, similar to that of the sea surface temperature climatology at both Neah Bay in the north [37] and Cape Elizabeth in the south [38]. During the NEP MHW years of 2014 and 2015, temperature anomalies at the OCNMS moorings at 40 m were as high as 4.5°C and lasted 10–20 days. There were also notable positive temperature anomalies during 2011 and 2013 (Fig 4). Analysis of Argo observations by Scannell et al. [20] indicates an earlier genesis of this NEP MHW with consistent warming observed as early as August 2013, suggesting that the September and October 2013 positive temperature anomalies were associated with the start of the NEP MHW, which Scannell et al. [20] labeled as the NEP 2013–2016 MHW. The positive temperature anomalies in 2011 are an exception to this, however, because no NEP MHW was observed during 2011. These observations suggest that the phenomenon of pulsed positive temperature anomalies is not uncommon along the Olympic Coast but may be accentuated by MHWs.

Understanding nearshore temperature variation helps us understand conditions to which marine organisms are exposed. Benthic organisms with limited motility may be particularly affected. A large body of research has been devoted to identifying thermal tolerances, particularly in intertidal and coastal environments [39–42]. Most marine species already live near the upper limit of their thermal tolerance [43, 44], and spatial and temporal variation in temperature in the marine environment can influence movement, migration, persistence and adaptation to climate change among marine organisms [45].

Notably, the upper 75 m of the ocean has warmed by an average of 0.11°C per decade from 1971–2010 globally [46]. This trend is expected to continue into the future [47, 48]. At the same time, the frequency, duration, and spatial extent of MHWs have increased in the last century [14, 49] and are expected to continue to increase, even under a warming scenario of as little as 1.5 ^**O**^C [49]. MHWs that occur on top of secular warming have the potential to push marine organisms beyond the upper bounds of their thermal tolerance.

Temperature also affects the solubility of dissolved oxygen in the ocean, which further constrains species survival. As temperatures increase, metabolic demand also increases, and hypoxia tolerance decreases [50]. Over time, the dual stressors of increasing temperature and declining oxygen are likely to force changes in the structure and function of marine communities, with consequences for nearshore ecosystems and the socio-economic benefits they provide.

We observed large positive temperature anomalies with abrupt onsets and magnitude much larger than the seasonal signal. These observations of nearshore temperature changes would not be detectable by satellite observations that have been to characterize MHWs in the past. Our findings that local winds were strongly correlated with the observed temperature anomalies suggest that similar phenomena could be operative in other upwelling systems. We further conclude that the temperature anomalies were directly related to upwelling and downwelling processes that drive vertical advection nearshore. Regional or local bathymetric or shoreline features specific to the Olympic region also may have played a role in the response of the nearshore environment to the winds during NEP 2013–2015 MHW. Yet, as the ocean continues to warm and MHWs become more common, we are likely to observe abrupt and large positive temperature anomalies more frequently. Such large and sudden temperature oscillations can affect biological and ecological processes, arguing for further investigation of nearshore responses to MHWs across upwelling systems.

## Conclusion

Effects of the 2013–2015 northeast Pacific Ocean MHW were widespread over the California Current System but were also spatially and temporally variable. This variability is particularly evident in the nearshore environment of Washington’s Olympic coast. Our results show that positive temperature anomalies that had a magnitude of about twice the size of the seasonal cycle, even when considering the fluctuation in timing of the fall transition. Positive temperature anomalies were episodic, with temperature fluctuating on timescales of days to weeks instead of weeks to months. Notably, positive temperature anomalies were not restricted to the period of the MHW, but the large scale MHW may have accentuated the magnitude and timing of the nearshore temperature extremes. Correlations of the positive temperature anomalies with local wind forcing, currents, and Ekman transport suggest that mechanisms of upwelling and downwelling likely caused much of the observed variability. Such large temperature excursions and their fluctuating nature could very well impact nearshore biota. Understanding the potential effects of such events on nearshore ecosystems can help guide climate change adaptation and underscores the need for continued monitoring of nearshore environments.

## References

[1] Hobday AJ, Alexander LV, Perkins SE, Smale DA, Straub SC, Oliver EC, et al. A hierarchical approach to defining marine heatwaves. Progress in Oceanography. 2016 Feb 1;141:227–38. doi: 10.1016/j.pocean.2015.12.014

[2] Scannell HA, Pershing AJ, Alexander MA, Thomas AC, Mills KE. Frequency of marine heatwaves in the North Atlantic and North Pacific since 1950. Geophysical Research Letters. 2016 Mar 16;43(5):2069–76. doi: 10.1002/2015GL067308

[3] Colloca F, Cardinale M, Maynou F, Giannoulaki M, Scarcella G, Jenko K, et al. Rebuilding Mediterranean fisheries: a new paradigm for ecological sustainability. Fish and fisheries. 2013 Mar;14(1):89–109. doi: 10.1111/j.1467-2979.2011.00453.x

[4] Mills KE, Pershing AJ, Brown CJ, Chen Y, Chiang FS, Holland DS, et al. Fisheries management in a changing climate: lessons from the 2012 ocean heat wave in the Northwest Atlantic. Oceanography. 2013 Jun 1;26(2):191–5.

[5] Chen K, Gawarkiewicz GG, Lentz SJ, Bane JM. Diagnosing the warming of the Northeastern US Coastal Ocean in 2012: A linkage between the atmospheric jet stream variability and ocean response. Journal of Geophysical Research: Oceans. 2014 Jan;119(1):218–27. doi: 10.1002/2013JC009393

[6] Pearce AF, Feng M. The rise and fall of the “marine heat wave” off Western Australia during the summer of 2010/2011. Journal of Marine Systems. 2013 Feb 1;111:139–56. doi: 10.1016/j.jmarsys.2012.10.009

[7] Bond NA, Cronin MF, Freeland H, Mantua N. Causes and impacts of the 2014 warm anomaly in the NE Pacific. Geophysical Research Letters. 2015 May 16;42(9):3414–20. doi: 10.1002/2015GL063306

[8] Amaya DJ, Miller AJ, Xie SP, Kosaka Y. Physical drivers of the summer 2019 North Pacific marine heatwave. Nature communications. 2020 Apr 20;11(1):1–9. doi: 10.1038/s41467-020-15820-w 32313028 PMC7171163

[9] Garrabou J, Coma R, Bensoussan N, Bally M, Chevaldonné P, Cigliano M, et al. Mass mortality in Northwestern Mediterranean rocky benthic communities: effects of the 2003 heat wave. Global change biology. 2009 May;15(5):1090–103. doi: 10.1111/j.1365-2486.2008.01823.x

[10] Cavole LM, Demko AM, Diner RE, Giddings A, Koester I, Pagniello CM, et al. Biological impacts of the 2013–2015 warm-water anomaly in the Northeast Pacific: winners, losers, and the future. Oceanography. 2016 Jun 1;29(2):273–85.

[11] Wernberg T, Bennett S, Babcock RC, De Bettignies T, Cure K, Depczynski M, et al. Climate-driven regime shift of a temperate marine ecosystem. Science. 2016 Jul 8;353(6295):169–72. doi: 10.1126/science.aad8745 27387951

[12] Smale DA, Wernberg T, Oliver EC, Thomsen M, Harvey BP, Straub SC, et al. Marine heatwaves threaten global biodiversity and the provision of ecosystem services. Nature Climate Change. 2019 Apr;9(4):306–12. doi: 10.1038/s41558-019-0412-1

[13] Oliver EC, Burrows MT, Donat MG, Sen Gupta A, Alexander LV, Perkins-Kirkpatrick SE, et al. Projected marine heatwaves in the 21st century and the potential for ecological impact. Frontiers in Marine Science. 2019 Dec 4;6:734. doi: 10.3389/fmars.2019.00734

[14] Oliver EC, Donat MG, Burrows MT, Moore PJ, Smale DA, Alexander LV, et al. Longer and more frequent marine heatwaves over the past century. Nature communications. 2018 Apr 10;9(1):1–2. doi: 10.1038/s41467-018-03732-9 29636482 PMC5893591

[15] Jacox MG. Marine heatwaves in a changing climate, Nature. 2019; 571, 485–487, doi: 10.1038/d41586-019-02196-1 31332352

[16] Sherman K. Adaptive management institutions at the regional level: the case of large marine ecosystems. Ocean & Coastal Management. 2014 Mar 1;90:38–49.

[17] Guo X, Gao Y, Zhang S, Wu L, Chang P, Cai W, et al. Threat by marine heatwaves to adaptive large marine ecosystems in an eddy-resolving model. Nature Climate Change. 2022 Feb;12(2):179–86. doi: 10.1038/s41558-021-01266-5 35757518 PMC7612885

[18] Di Lorenzo E, Mantua N. Multi-year persistence of the 2014/15 North Pacific marine heatwave. Nature Climate Change. 2016 Nov;6(11):1042–7. doi: 10.1038/nclimate3082

[19] Joh Y, Di Lorenzo E. Increasing coupling between NPGO and PDO leads to prolonged marine heatwaves in the Northeast Pacific. Geophysical Research Letters. 2017 Nov 28;44(22):11–663. doi: 10.1002/2017GL075930

[20] Scannell HA, Johnson GC, Thompson L, Lyman JM, Riser SC. Subsurface evolution and persistence of marine heatwaves in the Northeast Pacific. Geophysical Research Letters. 2020 Dec 16;47(23):e2020GL090548.

[21] Leising AW, Schroeder ID, Bograd SJ, Abell J, Durazo R, Gaxiola-Castro G, et al. State of the California Current 2014–15: Impacts of the Warm-Water" Blob". California Cooperative Oceanic Fisheries Investigations Reports. 2015;56.

[22] Mickett JB, Newton JA, Alford M. Coastal Ocean and Puget Sound Boundary Conditions: NW Washington coast water properties. *In* Moore SK, Wold R, Stark K, Bos J, Williams P, Dzinbal K, Krembs C, Newton JA, eds. *Puget Sound Marine Waters*: *2014 Overview*. 2015; 8.

[23] Piatt JF, Parrish JK, Renner HM, Schoen SK, Jones TT, Arimitsu ML, et al. Extreme mortality and reproductive failure of common murres resulting from the northeast Pacific marine heatwave of 2014–2016. PloS one. 2020 Jan 15;15(1):e0226087. doi: 10.1371/journal.pone.0226087 31940310 PMC6961838

[24] Jones T, Parrish JK, Peterson WT, Bjorkstedt EP, Bond NA, Ballance LT, et al. Massive mortality of a planktivorous seabird in response to a marine heatwave. Geophysical Research Letters. 2018 Apr 16;45(7):3193–202.

[25] McCabe RM, Hickey BM, Kudela RM, Lefebvre KA, Adams NG, Bill BD, et al. An unprecedented coastwide toxic algal bloom linked to anomalous ocean conditions. Geophysical Research Letters. 2016 Oct 16;43(19):10–366. doi: 10.1002/2016GL070023 27917011 PMC5129552

[26] Newton JA, Jimenez Urias M, Li L, Li L, O’Brien Beaumont K, Shao A, et al. *Summary and Recommendations of the Second Pacific Anomalies Science and Technology Workshop*, Seattle, 2016. http://www.nanoos.org/resources/anomalies_workshop/workshop2/docs/paw-2-report.pdf.

[27] Large WG, Pond S. Open ocean momentum flux measurements in moderate to strong winds. Journal of physical oceanography. 1981 Mar;11(3):324–36. doi: 10.1175/1520-0485(1981)011&lt;0324:OOMFMI&gt;2.0.CO;2

[28] Liu Y, Weisberg RH. Momentum balance diagnoses for the West Florida Shelf. Continental Shelf Research. 2005 Nov 1;25(17):2054–74.

[29] Hickey BM. The fluctuating longshore pressure gradient on the Pacific Northwest shelf: A dynamical analysis. Journal of physical oceanography. 1984 Feb 1;14(2):276–93.

[30] Allen JS, Smith RL. On the dynamics of wind-driven shelf currents. Philosophical Transactions of the Royal Society of London. Series A, Mathematical and Physical Sciences. 1981 Sep 24;302(1472):617–34.

[31] Chan F, Barth JA, Blanchette CA, Byrne RH, Chavez F, Cheriton O, et al. Persistent spatial structuring of coastal ocean acidification in the California Current System. Scientific Reports. 2017 May 31;7(1):1–7. 28566727 10.1038/s41598-017-02777-y PMC5451383

[32] Carbonel CA. Modelling of upwelling–downwelling cycles caused by variable wind in a very sensitive coastal system. Continental Shelf Research. 2003 Oct 1;23(16):1559–78. 10.1016/S0278-4343(03)00145-6.

[33] Austin JA, Lentz SJ. The inner shelf response to wind-driven upwelling and downwelling. Journal of Physical Oceanography. 2002 Jul;32(7):2171–93.

[34] Mickett JB, Newton JA, Alford M. Coastal Ocean and Puget Sound boundary Conditions: Coastal ocean, interannual comparison of water properties. *In* Moore SK, Stark K, Bos J, Williams P, Newton JA, Dzinbal K, eds. 2014. *Puget Sound Marine Waters*: *2013 Overview*. 6.

[35] Mickett JB Newton JA. Coastal Ocean and Puget Sound Boundary Condiditons: NW Washington coast water properties. *In* Moore SK, Wold R, Stark K., Bos J, Williams P, Dzinbal K, Krembs C, Newton JA, eds. 2016. *Puget Sound Marine Waters*: *2015 Overview*: 7–8.

[36] Bograd SJ, Schroeder I, Sarkar N, Qiu X, Sydeman WJ, Schwing FB. Phenology of coastal upwelling in the California Current. Geophysical Research Letters. 2009 Jan;36(1). doi: 10.1029/2008GL035933

[37] NANOOS.org. 2019a. NVS: Climatology. http://nvs.nanoos.org/Climatology?action=oiw:site:NDBC_46087:plots:1:water_temp. Accessed March 8 2020.

[38] NANOOS.org. 2019b. NVS: Climatology. http://nvs.nanoos.org/Climatology?action=oiw:site:NDBC_46041:plots:1:water_temp. Accessed March 8 2020.

[39] Madeira D, Narciso L, Cabral HN, Vinagre C. Thermal tolerance and potential impacts of climate change on coastal and estuarine organisms. Journal of Sea Research. 2012 May 1;70:32–41. doi: 10.1016/j.seares.2012.03.002

[40] Vinagre C, Dias M, Roma J, Silva A, Madeira D, Diniz MS. Critical thermal maxima of common rocky intertidal fish and shrimps—A preliminary assessment. Journal of sea research. 2013 Aug 1;81:10–2. doi: 10.1016/j.seares.2013.03.011

[41] Vinagre C, Leal I, Mendonça V, Flores AA. Effect of warming rate on the critical thermal maxima of crabs, shrimp and fish. Journal of thermal biology. 2015 Jan 1;47:19–25. doi: 10.1016/j.jtherbio.2014.10.012 25526650

[42] Pörtner HO, Bock C, Mark FC. Oxygen-and capacity-limited thermal tolerance: bridging ecology and physiology. Journal of Experimental Biology. 2017 Aug 1;220(15):2685–96. doi: 10.1242/jeb.134585 28768746

[43] Sunday JM, Bates AE, Dulvy NK. Thermal tolerance and the global redistribution of animals. Nature Climate Change. 2012 Sep;2(9):686–90. doi: 10.1038/nclimate1539

[44] Pinsky ML, Worm B, Fogarty MJ, Sarmiento JL, Levin SA. Marine taxa track local climate velocities. Science. 2013 Sep 13;341(6151):1239–42. doi: 10.1126/science.1239352 24031017

[45] Sunday JM, Howard E, Siedlecki S, Pilcher DJ, Deutsch C, MacCready P, et al. Biological sensitivities to high‐resolution climate change projections in the California current marine ecosystem. Global Change Biology. 2022 Oct;28(19):5726–40. doi: 10.1111/gcb.16317 35899628 PMC9542873

[46] Rhein M, Rintoul SR, Aoki S, Campos E, Chambers D, Feely RA, et al, 2013: Observations: Ocean. In: Stocker T, editor. Climate change 2013: the physical science basis: Working Group I contribution to the Fifth assessment report of the Intergovernmental Panel on Climate Change. Cambridge university press; 2014 Mar 24.

[47] Lima FP, Wethey DS. Three decades of high-resolution coastal sea surface temperatures reveal more than warming. Nature communications. 2012 Feb 28;3(1):1–3. doi: 10.1038/ncomms1713 22426225

[48] Alexander MA, Scott JD, Friedland KD, Mills KE, Nye JA, Pershing AJ, et al. Projected sea surface temperatures over the 21st century: Changes in the mean, variability and extremes for large marine ecosystem regions of Northern Oceans. Elementa: Science of the Anthropocene. 2018 Jan 1;6. doi: 10.1525/elementa.191

[49] Frölicher TL, Fischer EM, Gruber N. Marine heatwaves under global warming. Nature. 2018 Aug;560(7718):360–4. doi: 10.1038/s41586-018-0383-9 30111788

[50] Deutsch C, Ferrel A, Seibel B, Pörtner HO, Huey RB. Climate change tightens a metabolic constraint on marine habitats. Science. 2015 Jun 5;348(6239):1132–5. doi: 10.1126/science.aaa1605 26045435

